# Polycystic Ovary Syndrome Phenotype D Versus Functional Hypothalamic Amenorrhea With Polycystic Ovarian Morphology: A Retrospective Study About a Frequent Differential Diagnosis

**DOI:** 10.3389/fendo.2022.904706

**Published:** 2022-06-02

**Authors:** Klara Beitl, Didier Dewailly, Rudolf Seemann, Marlene Hager, Jakob Bünker, Daniel Mayrhofer, Iris Holzer, Johannes Ott

**Affiliations:** ^1^Clinical Division of Gynecological Endocrinology and Reproductive Medicine, Department of Obstetrics and Gynecology, Medical University of Vienna, Vienna, Austria; ^2^Faculty of Medicine Henri Warembourg, University of Lille, Lille Cedex, France; ^3^Department of Oral and Maxillofacial Surgery, Medical University of Vienna, Vienna, Austria

**Keywords:** functional hypothalamic amenorrhea, polycystic ovary syndrome, testosterone, sexual hormone binding globulin, luteinizing hormone, estradiol

## Abstract

The two most frequent causes of secondary amenorrhea are polycystic ovary syndrome (PCOS) and functional hypothalamic amenorrhea (FHA). Despite several studies showing differences in hormonal profile between these groups, the differential diagnosis remains challenging, in particular between FHA women with polycystic ovarian morphology (FHA-PCOM) and PCOS patients without hyperandrogenism (phenotype D, PCOS-D). In a retrospective case-control study, 58 clearly defined patients with FHA-PCOM were compared to 58 PCOS-D patients, matched 1:1 for age and BMI. Significantly higher levels of LH, estradiol, testosterone, and a higher luteinizing hormone (LH): follicle stimulating hormone (FSH) ratio as well as lower sexual hormone binding globulin (SHBG) levels were found in PCOS-D patients (*p<* 0.05). Optimized cut-off values for the prediction of FHA-PCOM were calculated by the Youden index. The highest sensitivity was found for an estradiol serum level <37.5 pg/mL (84.5%, 95% confidence interval, CI: 72.6-92.6), whereas a LH : FSH ratio <0.96 had the highest specificity (94.8, 95% CI: 85.6-98.9). A linear discriminant analysis including testosterone, SHBG and LH was able to correctly classify 87.9% of FHA-PCOM patients (bootstrap 95% CI: 80.2 - 94.0%). In conclusion, this model including serological parameters could be an easy and reliable tool to distinguish between FHA-PCOM and PCOS-D patients, especially in situations where the clinical profile is not obvious.

## Introduction

Secondary amenorrhea is quite common in women of reproductive age with a prevalence of 3-5%. Notably, the two most frequent causes are polycystic ovary syndrome (PCOS) and functional hypothalamic amenorrhea (FHA) (1). Thus, they are relevant differential diagnoses which can be a challenge for physicians, especially given the fact that a high rate of women with FHA reveal polycystic ovarian morphology (PCOM) of up to nearly 50% (2).

PCOM is one of the key features of PCOS according to the widely used Rotterdam criteria, where two out of three criteria have to be fulfilled which also include clinical and/or serological hyperandrogenism as well as oligo-/anovulation, the latter usually leading to oligo-/amenorrhea (3, 4). Moreover, it is also a major definition criterion according to the Androgen Excess Society (5). In contrast, according to the Endocrine Society, FHA should be defined by a menstrual cycle length persistently exceeding 45 days or amenorrhea >3 months, history of weight loss/vigorous exercise/stress, and the presence of hypogonadotropic hypoestrogenism (1).

Several highly accurate parameters for the differentiation between PCOS and FHA have already been reported and have been reviewed recently (5). These include the body mass index (BMI), levels of luteinizing hormone (LH), androgens, insulin, anti-Müllerian hormone (AMH) and sexual hormone binding globulin (SHBG), the progesterone withdrawal test as well as endometrial thickness as easily applicable tools in clinical routine.

As concluded by Phylactou et al. (5), the exclusion of other reasons for oligo-/amenorrhea is warranted in the definition criteria for both PCOS and FHA and there is no available test that is ultimately discriminating. Moreover, it has been mentioned that these diagnostic uncertainties also make the initial assignment to PCOS or FHA in studies more difficult (6). Thus, a precise definition of PCOS and FHA would be desirable in studies about this specific topic.

The main concern is that one might confuse PCOS without hyperandrogenism (Rotterdam phenotype D; PCOS-D) with FHA with PCOM (FHA-PCOM). However, all previous studies on this topic compared PCOS and FHA without a focus on these special subtypes. We chose two groups of clearly defined cases, namely strictly defined FHA-PCOM patients and age- and BMI-matched women with PCOS-D. Thereby, we aimed to evaluate the most apparent differences in serological patient characteristics and create a simple statistical tool, which should alleviate the differential diagnosis between FHA-PCOM and PCOS-D in more complex situations in the future.

## Methods

This retrospective case-control study was conducted at the Clinical Division of Gynecologic Endocrinology and Reproductive Medicine of the Medical University of Vienna, Austria. From January 2012 to April 2021. Data were included from 58 patients with FHA having PCOM, defined as follows: secondary amenorrhea for at least six months and a negative progestogen challenge test with context of weight loss, insufficient caloric intake, intense physical activity or notion of recent psychological stress, confirmed by a psychologic report. Pregnancy, hypothyroidism, and hyperprolactinemia and any organ-related pituitary dysfunction had to be excluded. The control group consisted of 58 PCOS phenotype D patients diagnosed based on the Rotterdam criteria (4), who had responded well to a progesterone challenge test, and were matched 1:1 by age and BMI for all further analyses in this study. PCOS-D is one of four different phenotypes of PCOS and is also known as “non-hyperandrogenic” PCOS. It is characterized by oligo-/anovulation and PCOM (3). Since the definition of Androgen Excess Society would require hyperandrogenism as a mandatory criterion for PCOS diagnosis (5), the Rotterdam criteria were chosen. This classification has been recently re-visited and validated (3). In all patients, an Aloka Prosound 6 ultrasound machine (Wiener Neudorf, Austria; frequency range 3.0 – 7.5 MHz) was used. PCOM was defined by a follicle number per ovary (FNPO) >12 and/or an ovarian volume ≥10 cm3 and/or an ovarian area ≥5.5 cm^2^, according to the recommendations of an international expert panel for ultrasound machine with frequency range less than 8 MHz (7).

The study protocol complies with the declaration of Helsinki and was approved by the Institutional Review Board of the Medical University of Vienna (institutional review board number 1722/2021). Neither written nor verbal informed consent was necessary in retrospective studies according to the Ethics Committee of the Medical University of Vienna.

### Parameters Analyzed

As the main outcome parameter, we focused on serum levels of AMH. Additionally, serum levels of total testosterone, androstenedione, SHBG, LH, follicle-stimulating hormone (FSH) and estradiol were also analyzed. The AKIM-software (SAP-based patient management system; SAP Software Solutions Austria, Vienna, Austria) was used for data acquisition.

Blood samples were obtained from a peripheral vein on cycle days 2-5 after bleeding induction with oral dydrogesterone (see below), if possible, or during amenorrhea if no menstruation could be induced with dydrogesterone. All examined blood parameters were determined at the Department of Laboratory Medicine, General Hospital of Vienna, Vienna, Austria according to ISO 15189 quality standards: estradiol, follicle-stimulating hormone (FSH), luteinizing hormone (LH), anti-Mullerian hormone (AMH) and sex hormone-binding globulin (SHBG) were measured by the corresponding Cobas electrochemiluminescence immunoassays (ECLIA) on Cobas e 602 analyzers (Roche, Mannheim, Germany).

The following basic patient characteristics were also included: age at evaluation, body mass index (BMI), gravidity and parity.

### Statistical Analysis

Categorical parameters are presented as numbers and frequencies, continuous data as median and their respective interquartile range (IQR). Mann-Whitney U tests were used to compare independent continuous variables, Wilcoxon rank sum test for dependent continuous variables. Categorical variables between two groups were compared by chi-square or Fisher´s exact test. Receiver operator characteristic (ROC) curves were computed to determine the sensitivity and specificity of serum parameters for FHA-PCOM and help to find optimal cut-off points which were then defined by the Youden index. For these optimized cut-off values, sensitivity, specificity, positive (PPV) and negative predictive values (NPV) with their according 95% confidence intervals (95% CI) are provided. Linear discriminant analyses (LDA) were performed to find linear classifiers separating both groups using FSH, LH, Estradiol, Testosterone, SHBG, and AMH. A confidence interval for the percentage of right classified patients was found by generating 500 bootstrap replicates. Statistical significance was defined by two-sided *P*-values <0.05. Statistical analyses were performed using the open source software “R” (R: The R Project for Statistical Computing).

## Results

### Basic Patient Characteristics

Due to the matching for age and BMI, these parameters did not differ between women with FHA-PCOM and women with PCOS-D. Concerning hormonal findings, patients with FHA-PCOM revealed significantly higher levels of SHBG. In contrast, significantly higher levels of LH, estradiol, testosterone, androstenedione, DHEAS, and prolactin as well as a higher LH : FSH ratio were found in PCOS-D patients. Details are shown in **Table 1**.

**Table 1:** Basic patient characteristics and results of hormonal testing in FHA-PCOM and PCOS-D patients. Data are presented as mean ± SD.

	FHA-PCOM	PCOS-D	p
Age (years)	25.5 ± 4.7	25.5 ± 4.7	1.000
BMI (kg/m^2^)	26.4 ± 6.3	26.3 ± 6.2	0.983
TSH (IU/mL)	1.7 ± 0.7	2.0 ± 1.0	0.053
FSH (mIU/mL)	5.2 ± 2.1	5.1 ± 1.9	0.930
LH (mIU/mL)	3.6 ± 3.1	8.8 ± 5.6	<0.001
LH : FSH ratio	0.7 ± 0.5	1.7 ± 1.0	<0.001
Prolactin (ng/mL)	10.2 ± 4.1	13.5 ± 7.7	0.036
Estradiol (pg/mL)	23.0 ± 14.1	53.3 ± 19.4	<0.001
Testosterone (ng/mL)	0.22 ± 0.12	0.38 ± 0.09	<0.001
Androstenedione (ng/mL)	2.0 ± 1.1	2.7 ± 0.9	0.003
DHEAS (µg/mL)	2.2 ± 1.1	2.5 ± 0.8	0.048
SHBG (nmol/L)	81.8 ± 41.8	49.8 ± 37.4	<0.001
AMH (ng/mL)	6.9 ± 3.8	8.4 ± 5.1	0.071

### Optimized Cut-Off Values

The ROC curves of the tested serum parameters for FHA-PCOM are shown in the **Supplementary Figure 1**. Only estradiol, testosterone, SHBG, LH, and the LH : FSH ratio were found to be significantly predictive for FHA-PCOM (*p<* 0.05). **Table 2** shows the optimized thresholds for these values calculated by the Youden index. The highest sensitivity was found for estradiol <37.5 pg/mL (84.5%, 95% CI: 72.58-92.65), whereas as a LH : FSH ratio <0.96 had the highest specificity (94.8, 95% CI: 85.6-98.9).

**Table 2:** Optimized cut-off values for FHA-PCOM.

Parameter	Statistical method	Optimized cut-off value	Sensitivity (95%CI)	Specificity (95%CI)	PPV (95%CI)	NPV (95%CI)	p
Estradiol	Youden index	<37.5 pg/mL	84.48 (72.58-92.65)	82.76 (70.57-91.41)	83.05 (73.39-89.69)	84.21 (74.31-90.77)	<0.001
Testosterone	Youden index	<0.31 ng/mL	79.31 (66.65-88.83)	86.21 (74.21-93.85)	85.19 (74.89-91.73)	80.65 (71.36-87.45)	<0.001
SHBG	Youden index	>61.4 nmol/L	68.97 (55.46-80.46)	79.31 (66.65-88.83)	76.92 (66.18-85.03)	71.88 (63.01-79.31)	<0.001
LH	Youden index	<4.7 mIU/mL	74.14 (60.69-84.74)	77.59 (64.73-87.49)	76.79 (66.68-84.54)	75.00 (65.51-82.57)	<0.001
LH: FSH ratio	Youden index	<0.96	72.41 (59.10-83.34)	94.83 (85.62-98.92)	93.33 (82.14-97.71)	77.46 (96.28-83.97)	<0.001

All data are provided as %; PPV, positive predictive value; NPV, negative predictive value.

### Linear Discriminant Models

In separate linear discriminant models using only one feature, the rates of correctly classified patients were 81.7% for estradiol, 81.7% for testosterone, 71.5% for SHBG, 71.3% for LH, 68.8% for LH : FSH ratio, 55.0% for FSH, and 56.6% for AMH. In **Table 3**, results of the linear discriminant analyses are shown. A linear discriminant analysis incorporating all serologic features, namely estradiol, testosterone, SHBG, LH, the LH : FSH ratio, FSH, and AMH (“full model”), correctly classified 92.9% of the patients (bootstrap 95% CI: 85.3 - 98.2%).

**Table 3:** Results of the linear discriminant analyses.

Parameter	FHA-PCOM	PCOS-D	Coefficients of linear discriminants		
			Full model	Reduced model 1	Reduced model 2
Estradiol (pg/mL)	23.0 ± 14.1	53.3 ± 19.4	0.039	0.004	
Testosterone (ng/mL)	0.22 ± 0.12	0.38 ± 0.09	4.687	5.108	7.050
SHBG (nmol/L)	81.8 ± 41.8	49.8 ± 37.4	-0.006	-0.009	-0.005
LH (mIU/mL)	3.6 ± 3.1	8.8 ± 5.6	0.012		0.117
LH : FSH ratio (mIU/mL)	0.7 ± 0.5	1.7 ± 1.0	0.414		
FSH (mIU/mL)	5.2 ± 2.1	5.1 ± 1.9	0.060		
AMH (ng/mL)	6.9 ± 3.8	8.4 ± 5.1	0.035		
Constant			-3.637	-2.601	-2.463

In clinical routine, easily applicable tools must be available. Thus, two “reduced models” were calculated. The “reduced model 1” included only the three strongest parameters estradiol, testosterone and SHBG and was able to correctly classify 91.3% of the patients (bootstrap 95% CI: 84.5 - 96.6%). However, one of the main criteria to assign patients in either the FHA-PCOM or PCOS-D group was whether menstruation could be induced by a gestagen withdrawal test (see Methods Section), which is known to be strongly correlated to serum estradiol levels. Since the aim was to generate a model using serum parameters which should help to distinguish between the two entities in more complex situations, a “reduced model 2” without estradiol was calculated. This model included the features testosterone, SHBG, and LH and correctly classified 87.9% of patients (bootstrap 95% CI: 80.2 - 94.0%).

**Figure 1:**
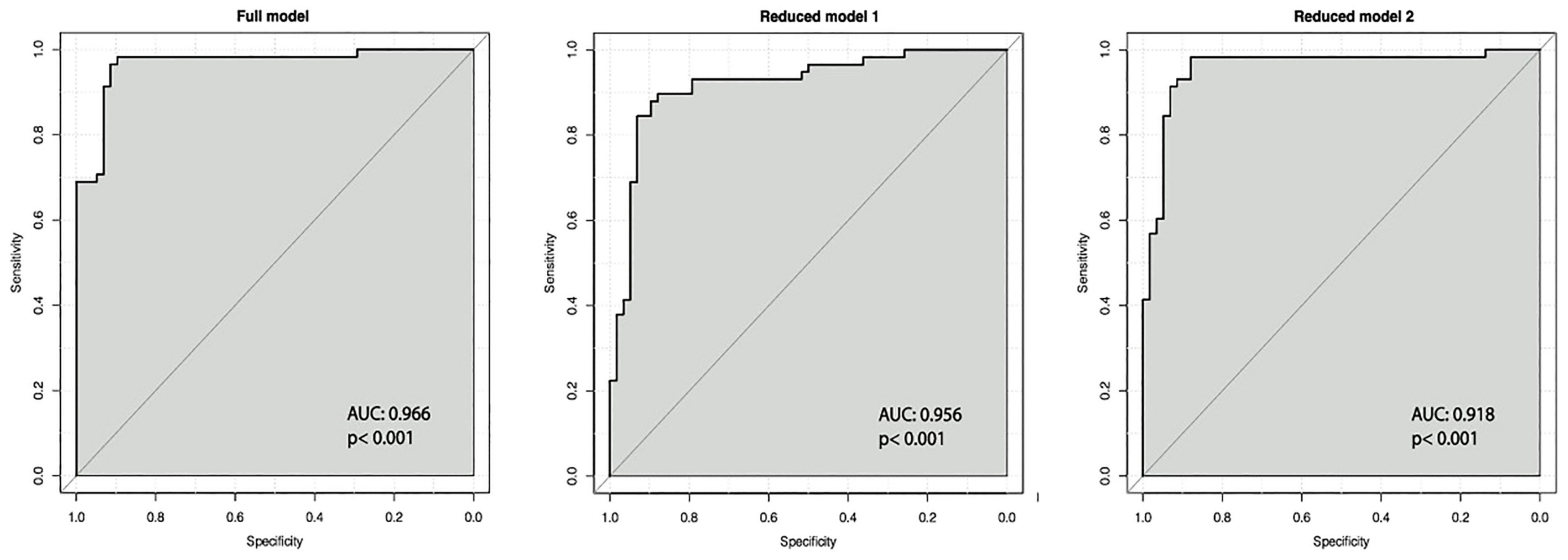
ROC curves for linear discriminant models. For each parameter, the area under the curve (AUC) and the p-value are provided.

The ROC curves for all these three models are provided in **Figure 1**.

### Practical Application of the Linear Discriminant Models

**Figure 2:**
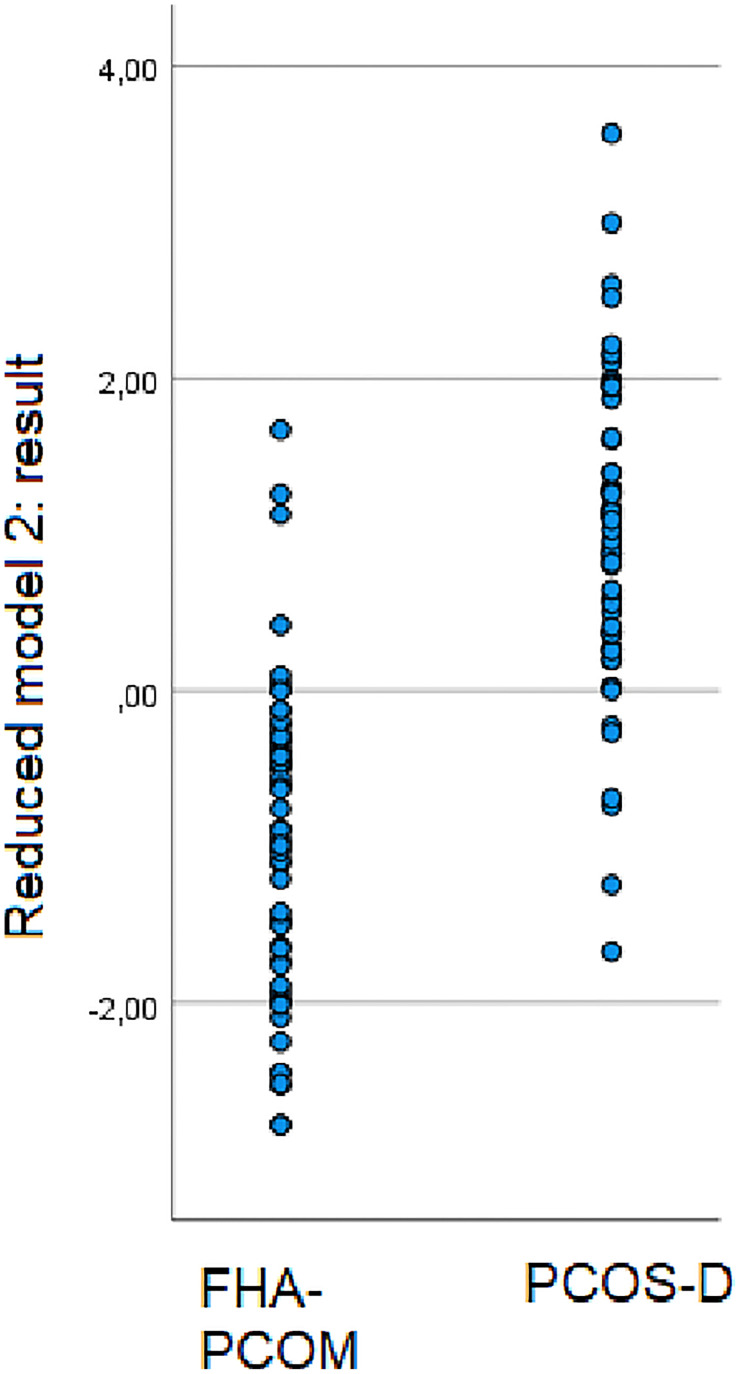
The reduced linear discriminant analysis includes testosterone, SHBG and LH as predictive parameters for FHA-PCOM. The scatter plot shows the results of the formula used “(7.05*testosterone ng/mL) – (0.005*SHBG nmol/L) + (0.117*LH mIU/mL) - 2.463” for women with FHA-PCOM and PCOS-D.

The “reduced model 2” (**Table 2**) is simple to use. The linear combination of the weighted features is either ≤0, whereby the patient belongs to the group of FHA-PCOM patients, or >0, whereby the patient belongs to the group of patients of PCOS-D. The following formula must be used: (7.05*testosterone ng/mL) – (0.005*SHBG nmol/L) + (0.117*LH mIU/mL) - 2.463. A scatter plot showing the results of this calculation for women with FHA-PCOM and women with PCOS-D is provided in **Figure 2**. The following predictive values for FHA-PCOM using the mentioned cut-off point of ≤0 were: sensitivity 87.9% (95% CI: 76.7-95.0), specificity 89.7% (95% CI: 78.8-96.1), positive predictive value 89.5% (95% CI: 79.8-94.8), and negative predictive value 88.1% (95% CI: 78.7-93.7; p< 0.001).

In case of patient number 1, who belongs to the group of women with FHA-PCOM, the patient had a testosterone level of 0.3 ng/mL, SHBG of 69.1 nmol/L and LH of 2.1 mIU/mL. The linear classifier is computed as:


(7.05∗0.30)–(0.005∗69.1)+(0.117∗2.1)−2.463=−0.4478

The weighted sum is below zero and therefore the patient belongs to the group of FHA-PCOM women.

In contrast, patient number 2 who belongs to the group of PCOS-D women, revealed the following serum parameters: testosterone 0.44 ng/mL, SHBG 131.90 nmol/L, and LH 17.2 mIU/mL. In this case, the linear classifier is computed as:


(7.05∗0.44)–(0.005∗131.9)+(0.117∗17.2)−2.463=1.9919

The weighted sum is greater than zero and, thus, the patient is allocated to the group of women with PCOS-D.

## Discussion

To distinguish women with FHA-PCOM from women with PCOS-D, the present study revealed that the following parameters would be useful: testosterone, LH, the LH : FSH ratio, and SHBG. Using optimized cut-off values calculated by the Youden index, the sensitivity ranged from about 72% to about 79% (**Table 2**). Including these features in linear discriminant analyses, even a “reduced model” using only a minority of accurate parameters, 87.9% of patients could be classified correctly.

We decided to exclude estradiol from our discriminating variables, although its level was significantly lower in women with FHA-PCOM than in PCOS-D patients, a fact that has been reported many times (8). The reason for this is that in our study, a progesterone withdrawal test was used to assign patients in either the FHA-PCOM or PCOS-D group. This was done to define the two groups in the best way possible. Therefore, highly significantly declined estradiol levels were observed in our FHA group, but this result was obviously biased, due to our methodical approach. Another reason for not using estradiol is that previous articles on women with FHA (2, 9) suggested intermittent estrogen production and, thus, levels within the normal range. Therefore, the data about estradiol in our model with well-defined cases should not be used to better distinguish between patients, where assignment to one or the other group is not similarly obvious.

This leads to the question of how patients were assigned to the groups. Any methodical approach to this question can be considered problematic. However, strict definition criteria of FHA-PCOM were defined: in addition to the negative progesterone challenge test, a cause for FHA had to be evident, namely weight loss, insufficient caloric intake, intense physical activity or notion of recent psychological stress. Thus, it seems very likely that all women classified as “FHA-PCOM” actually suffered from this entity. However, one could see the definition of PCOS- D as problematic and might assume that some FHA-PCOM patients may have been allocated to this group incorrectly. We consider these circumstances as a study limitation. However, based on the strict criteria, we believe that we have been able to define the groups in the best way possible.

Apart from estradiol, testosterone showed the highest sensitivity for the diagnosis of FHA-PCOM, with an optimized cut-off value of 0.31 ng/mL. This was associated with a high specificity of about 86%, which was also reflected by the high PPV of 85.2 and NPV of 80.6% (**Table 2**). These results show the importance of testosterone despite the fact that per definition, patients with PCOS-D do not have clinical or serological hyperandrogenemia. Nevertheless, testosterone was significantly higher in PCOS-D patients than in women with FHA-PCOM, although being in the normal range (**Table 1**). Since the ovaries are a main source for testosterone production in women (10) and ovarian function is limited in FHA (2), it seems intuitive that lower testosterone levels are found in FHA patients.

Moreover, in our analysis, the LH : FSH ratio with a cut- off value of <0.96 has been shown to be a strong predictor, very reliably predicting FHA-PCOM for the individual patient with a PPV of 93.3% (**Table 2**). It is known that PCO patients tend to have an increased LH : FSH ratio (11), while on the other hand FHA patients have lower LH levels (2, 9). Together with the significantly lower and also highly predictive LH levels (**Tables 1** and **2**), our results are consistent with the existing literature. One might argue that the LH : FSH ratio of 1.7 found in our PCOS patients would not be typical. However, it has been reported that testosterone levels would be positively correlated with the LH : FSH ratio (12). Since only PCOS-D women without hyperandrogenism were included, the comparably lower mean LH : FSH ratio would be reasonable.

Contrary to our expectations, the prognostic potential of SHBG, previously reported as a promising parameter to distinguish between FHA and PCOS (6), was comparably moderate. It is known that PCOS patients reveal lower SHBG levels (13). However, a recent analysis by Makollé et al. (2) already showed that women with FHA-PCOM might have a tendency to metabolic aspects of the PCO syndrome. Moreover, in this study, it became evident that patients with FHA-PCOM revealed lower SHBG levels than FHA women without PCOM (2). This could explain why SHBG proved to be weaker in prediction of FHA, although a PPV of approximately 77% and a NPV of 72% can be considered relatively reliable for a single parameter. **Table 1** displays wide variations of SHBG levels in both groups. It should be mentioned that women with FHA and underweight and/or eating disorders are known to have very high SHBG levels (14, 15).

In contrast to all these parameters, the ROC analyses showed that AMH was not predictive (**Supplementary Figure 1**). Recently, it has been described that especially women with FHA-PCOM tended to have high AMH levels, compared to non-PCOM FHA patients (2), which explained previous findings (9, 16). It has been hypothesized that FHA-PCOM patients initially exhibit components of PCOS before subsequently developing FHA due to weight loss, insufficient caloric intake, stress, or excessive exercise. Conclusive for this hypothesis is also a higher body mass index (BMI) and lower levels of sex hormone binding globulin (SHBG) in FHA-PCOM patients with PCOM compared to non-PCOM FHA women (2).

For this reason, AMH was not included in the calculations of the reduced linear discriminant models (**Table 3**). We believe that especially the reduced model 2, which did not include estradiol due to the above-mentioned methodical considerations, might be the most relevant and clinically applicable one. This model can be used to enable a well-founded differential diagnosis in less clearly defined cases. It offers equally high PPV and NPV of 89.5% and 88.1%, respectively. The majority of FHA-PCOM cases could be classified correctly (87.9%), which is also underlined by the small confidence interval in the bootstrap analysis (80.2 - 94.0%). In absence of any other clear criteria to distinguish the two entities from each other, we consider this approach helpful. We are aware of the fact that future studies are needed to clarify whether the model is correct and probably adjust it.

Concerning possible study limitations, the above-mentioned definition criteria of the two groups must be considered once more. In addition to the already mentioned considerations, one might wonder whether the included FHA population consisted in fact of PCOS-D patients who had hypogonadotropic stress and were recruited after partial recovery. Although this cannot be ruled out completely, it has already been shown that women with well-defined FHA-PCOM were not at an increased risk for developing PCOS in the course of pulsatile GnRH therapy (17). Moreover, only in a minority of FHA women, PCOS becomes unmasked by pulsatile GnRH treatment (18). Thus, we assume that it is unlikely that there was a majority of PCOS-D patients who were unintentionally allocated to the FHA-PCOM group.

Although the model is proposedly useful to help clinicians with an easy tool to distinguish between FHA-PCOM and PCOS-D in less clearly defined cases, it may not be transferable to all patients in every case. In addition, the retrospective study design and the small sample size must certainly be mentioned as study limitations. On the other hand, to our knowledge, this is the first study to attempt to discriminate between two groups which are likely very difficult to distinguish from each other, namely FHA-PCOM and PCOS-D, under optimally defined criteria. Moreover, one might argue that the mean BMI of about 26 kg/m^2^ is unusual for women with FHA who often suffer from underweight and eating disorders. It should be emphasized that we matched both groups on BMI, which obviously resulted to select FHA-PCOM patients with normal or slightly elevated BMI. It is precisely those patients who are at risk to be diagnosed PCOS-D.,Since we chose to use strict criteria for the definition of FHA, many women with intense physical activity were included. These often reveal a normal BMI due to the high muscle mass. In addition, the notion of recent psychological stress in FHA women is also not necessarily associated with a low BMI. Nonetheless, this circumstance should be considered a minor study limitation. In addition, the mean AMH levels of 6.9 ng/mL could be considered high for FHA patients. However, similar levels have been reported previously. For example, in a cluster analysis, 48% of FHA patients revealed PCOM. In a cluster with 70% of women with PCOM, the median AMH was 60.6 pmol/L, which is equal to 8.48 ng/mL (9). Many other publications showed normal or increased AMH levels in FHA patients despite low FSH and LH levels (9, 19, 20). Further studies even reported significant differences in AMH levels comparing FHA patients to controls (16, 21, 22).

In conclusion, clinical and serological differentiation between FHA-PCOM and PCOS-D can be challenging. A combination of low testosterone levels, a low LH : FSH ratio, and a higher SHBG level yielded the strongest predictive value for FHA, compared to any of the most discriminant variables used alone. The formula “(7.05*testosterone ng/mL) – (0.005*SHBG nmol/L) + (0.117*LH mIU/mL) - 2.463” can be used as an easy tool for this differential diagnosis. Further studies would be desirable to shed more light on this challenging topic.

## Data Availability Statement

The original contributions presented in the study are included in the article/**Supplementary Material**. Further inquiries can be directed to the corresponding author.

## Ethics Statement

The studies involving human participants were reviewed and approved by the Institutional Review Board of the Medical University of Vienna (institutional review board number 1722/2021). Written informed consent was not required for this study, in accordance with the local legislation and institutional requirements.

## Author Contributions

KB: protocol/project development, data collection or management, data analysis, manuscript writing/editing, final proof-reading. DD: protocol/project development, data management, data analysis, manuscript writing/editing, final proof-reading. RS: protocol/project development, data management, data analysis, manuscript writing/editing, final proof-reading. MH: protocol/project development, data collection or management, manuscript writing/editing, final proof-reading. JB: protocol/project development, data collection, final proof-reading. DM: protocol/project development, data collection or management, final proof-reading. IH: protocol/project development, manuscript writing/editing, final proof-reading. JO: protocol/project development, data collection or management, data analysis, manuscript writing/editing, final proof-reading. All authors contributed to the article and approved the submitted version.

## Conflict of Interest

The authors declare that the research was conducted in the absence of any commercial or financial relationships that could be construed as a potential conflict of interest.

## Publisher’s Note

All claims expressed in this article are solely those of the authors and do not necessarily represent those of their affiliated organizations, or those of the publisher, the editors and the reviewers. Any product that may be evaluated in this article, or claim that may be made by its manufacturer, is not guaranteed or endorsed by the publisher.

## Supplementary Material

Supplementary Figure 1 *ROC curves for FHA-PCOM*. For each parameter, the area under the curve (AUC) and the p-value are provided.
