# Supplementary figures and images for: Polycystic Ovary Syndrome Phenotype D Versus Functional Hypothalamic Amenorrhea With Polycystic Ovarian Morphology: A Retrospective Study About a Frequent Differential Diagnosis
Source: Front Endocrinol (Lausanne). 2022 Jun 2;13:904706. doi: 10.3389/fendo.2022.904706 (PMC9201247; doi:10.3389/fendo.2022.904706)

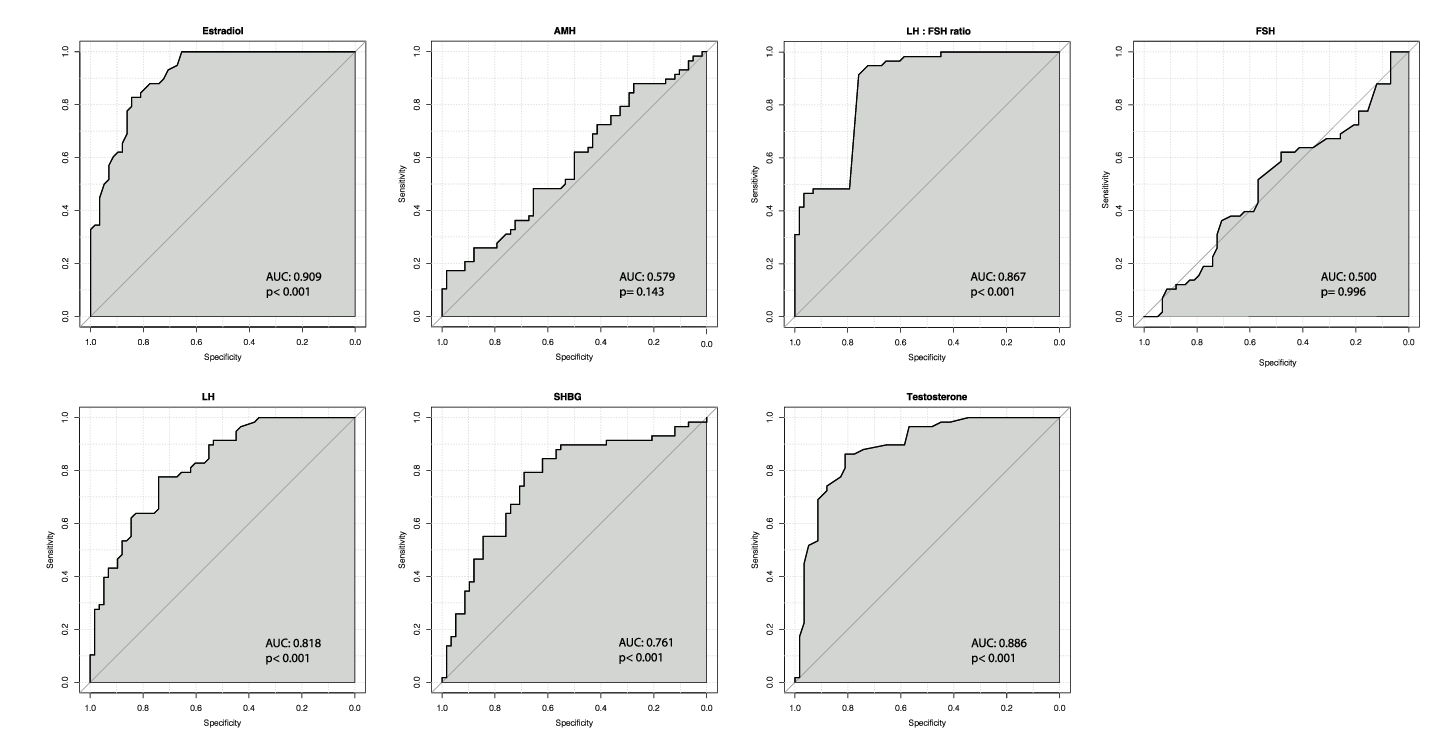

Supplement: Supplementary Figure 1 — ROC curves for FHA-PCOM. For each parameter, the area under the curve (AUC) and the p-value are provided. [file Image_1.tif]
